# Global Myocardial Work-Derived Nomogram for Coronary Stenosis Assessment in Stable Coronary Artery Disease: Development and External Validation

**DOI:** 10.3390/diagnostics16040570

**Published:** 2026-02-13

**Authors:** Miao Li, Wenfang Wu, Lin Li, Qianshan Ding, Jing Dong, Pingyang Zhang

**Affiliations:** Department of Cardiovascular Ultrasound, Nanjing First Hospital, Nanjing Medical University, 68 Changle Road, Nanjing 210006, China; lim0806@foxmail.com (M.L.); maomao864@126.com (W.W.);

**Keywords:** coronary artery disease, myocardial work efficiency, non-invasive assessment, nomogram, risk stratification

## Abstract

**Background:** Non-invasive identification of coronary stenosis in stable coronary artery disease (CAD) patients lacking regional wall motion abnormalities (RWMA) remains challenging. This study aimed to develop and validate a myocardial work-derived nomogram for predicting significant coronary stenosis in these patients. **Methods:** In this retrospective study, 181 consecutive patients with angiographically confirmed CAD, preserved LVEF (≥55%), and no resting wall motion abnormalities were enrolled. Global myocardial work efficiency (GWE) was assessed using echocardiographic pressure–strain loop analysis. A multivariable-derived nomogram incorporating GWE and clinical biomarkers was developed and externally validated for predicting severe coronary stenosis. **Results:** The nomogram incorporating GWE, lipoprotein-associated phospholipase A2 (LP-PLA2), N-terminal pro brain natriuretic peptide (NT-proBNP), and serum creatinine (Scr) demonstrated favorable discrimination in both the training set (AUC 0.916, 95% CI 0.866–0.952) and validation set (AUC 0.911, 95% CI 0.853–0.951), with good calibration (mean absolute error: 1.9% vs 3.2% in training vs validation, respectively). Decision curve analysis confirmed clinical utility across all probability thresholds. **Conclusions:** Our nomogram provides a non-invasive tool for preoperative risk stratification and optimizes the use of invasive diagnostics in stable CAD patients without RWMA.

## 1. Introduction

Coronary artery disease (CAD) accounts for the majority of cardiovascular mortality [1,2]. Early detection of CAD lesions is critical for optimizing treatment strategies and improving prognosis. While invasive coronary angiography (CAG) is established as the diagnostic reference standard for defining CAD severity [3], its procedural risks, contrast-related complications, and radiation exposure pose significant constraints on routine application [4]. Moreover, a large-scale study revealed that only 38% of stable patients suspected of having CAD exhibited significant obstructive lesions detectable by CAG [5]. These findings underscore the need for improved risk stratification strategies to guide clinical decision-making and enhance the diagnostic yield of cardiac catheterization in routine practice.

Non-invasive methods have shown promise in significantly improving the detection rate of CAG, particularly in patients with stable CAD [6]. Conventional transthoracic two-dimensional echocardiography is a widely utilized non-invasive technique in clinical practice for CAD detection, primarily relying on the assessment of reduced left ventricular ejection fraction (LVEF) and the identification of regional wall motion abnormalities (RWMA). However, due to the presence of collateral circulation and other compensatory mechanisms, RWMA may be absent, and LVEF may remain preserved even in patients with severe triple-vessel disease.

For patients with preserved LVEF and normal resting wall motion, speckle-tracking echocardiography (STE) offers a promising non-invasive alternative for stenosis detection [7,8]. Nevertheless, the diagnostic reliability of this technique is undermined by its inherent load dependence, posing a significant limitation to its routine clinical use [9].

Myocardial work (MW) analysis, a novel non-invasive technique, integrates myocardial strain and afterload, thereby enabling a comprehensive quantification of cardiac mechanical function [10]. Studies have demonstrated that left ventricular MW assessment is more sensitive than traditional indicators in detecting early myocardial dysfunction [11,12,13,14,15,16].

In our previous study, we demonstrated that global work efficiency (GWE) is the optimal parameter for predicting severe coronary stenosis in CAD patients without RWMA, underscoring its potential as a predictive indicator for identifying severe coronary artery disease [17]. However, the evaluation efficacy and clinical utility of the single parameter are limited. To translate this parameter into a robust clinical tool, we sought to develop and validate an integrative nomogram prediction model for severe coronary stenosis in CAD patients without RWMA by integrating GWE with established clinical indicators.

## 2. Materials and Methods

### 2.1. Clinical Enrollment

**Figure 1:**
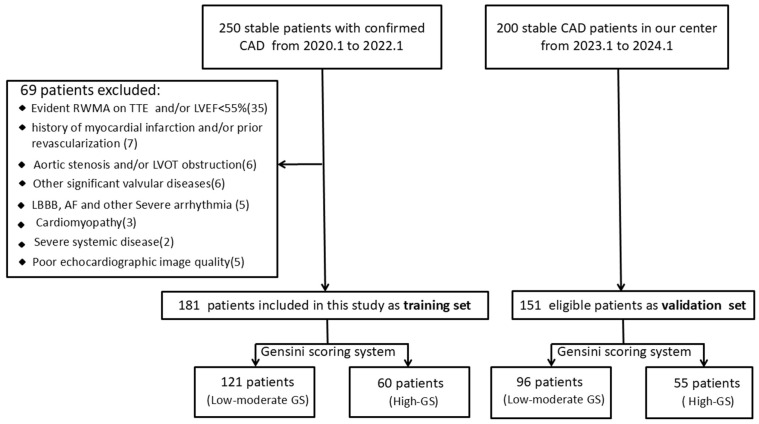
Flow chart of patient enrollment.

This retrospective study included a cohort of 250 patients diagnosed with confirmed CAD by CAG at Nanjing First Hospital between January 2020 and January 2022. The study cohort was restricted to clinically stable patients. Individuals with suspected acute coronary syndrome, prior myocardial infarction, or a history of revascularization were excluded. All participants underwent transthoracic echocardiography (TTE) within 12 h before coronary angiography (CAG). Further exclusion criteria were evident regional wall motion abnormalities (RWMA) or left ventricular ejection fraction (LVEF) <55% on TTE, left-sided obstructive lesions (aortic stenosis or LVOT obstruction), significant valvular disease, cardiomyopathy, severe arrhythmia, poor echocardiographic image quality, and other major systemic disorders (see Figure 1 for details). Following the screening process, 181 patients constituted the training cohort. An independent validation cohort was formed by 151 consecutive eligible patients from our center, recruited between January 2023 and January 2024.

The study was conducted in accordance with the principles of the Declaration of Helsinki and was approved by the Ethics Committee of Nanjing First Hospital (protocol code KY-20200110-03; date of approval 10 January 2020). Written informed consent was obtained from all participants prior to their inclusion in the study.

### 2.2. Clinical Data

Baseline clinical characteristics were retrospectively collected from electronic hospital records, encompassing sex, age, heart rate (HR), body mass index (BMI), systolic blood pressure (SBP), diastolic blood pressure (DBP), smoking status, history of hypertension, history of hyperlipidemia, history of diabetes mellitus, medications, total cholesterol (TC), triglycerides (TG), low-density lipoprotein (LDL), high-density lipoprotein (HDL), apolipoprotein A1 (ApoA1), apolipoprotein B (ApoB), lipoprotein A (LP-A), serum creatinine (Scr), blood glucose (BG), lipoprotein phospholipase A2 (LP-PLA2) and N-terminal pro brain natriuretic peptide (NT-proBNP).

### 2.3. Coronary Atherosclerosis Risk Stratification

In this study, coronary atherosclerosis risk stratification was assessed using the Gensini scoring system derived from coronary angiography. All coronary angiograms were independently analyzed by two interventional cardiologists blinded to the patients’ clinical and echocardiographic data. The Gensini scoring system was used to evaluate the severity of coronary artery stenosis [18,19]. The Gensini score (GS) was calculated through a two-step process: (1) assigning standardized severity scores to individual coronary stenoses, with adjusted weighting for total occlusions or 99% obstructive lesions supported by collateral circulation, followed by (2) applying a location-specific coefficient reflecting the hemodynamic significance of each lesion’s position within the coronary arterial tree [18] (detailed in Supplementary Table S1).

In instances of discordant assessment, a third independent interventional cardiologist, blinded to all ancillary data and study protocol, adjudicated the angiograms to determine the final Gensini score (GS). A GS > 0 defined the presence of CAD. Subsequently, patients with angiographically confirmed CAD were stratified into low-, moderate-, and high-GS subgroups based on tertile distribution of the Gensini scores.

### 2.4. Two-Dimensional Transthoracic Echocardiography

Standard two-dimensional TTE examinations were conducted in accordance with the American Society of Echocardiography recommendations [20]. All participants underwent continuous electrocardiographic monitoring throughout the procedure. The examinations were performed within 12 h preceding CAG using a Vivid E95 ultrasound system (GE Healthcare, Horten, Norway) equipped with an M5S transducer (3.5 MHz). Stroke volume (SV) and LVEF (%) were calculated using the modified bi-plane Simpson method.

### 2.5. Global Longitudinal Strain and Myocardial Work Analysis

Standardized offline analysis involved acquiring images in standard apical views (long-axis, two-chamber, and four-chamber), each containing three consecutive cardiac cycles. A subsequent independent review was performed by two experienced sonographers blinded to both the patients’ clinical information and each other’s assessments. To optimize myocardial deformation analysis, image acquisition was maintained at frame rates between 50 and 80 frames per second. Left ventricular global longitudinal strain (GLS) was assessed through semi-automated speckle-tracking analysis (EchoPAC software v204, GE Healthcare). Valve event timing was verified by visual inspection of the apical long-axis view before strain computation. An automatic algorithm performed the initial tracing and tracking of the left ventricular (LV) myocardium, with manual corrections applied to the endocardial border as needed. GLS values were derived from the weighted mean of segmental peak longitudinal strain measurements.

Myocardial work (MW) parameters were computed using dedicated analysis software (EchoPAC version 204, GE Healthcare). The methodology of MW measurement derived from non-invasive pressure–strain loops (PSLs) has been described previously [9]. Peak systolic left ventricular pressure was approximated by brachial artery sphygmomanometry (Omron, Kyoto, Japan) during echocardiographic acquisition. The software algorithm generated adjusted LV pressure curves by incorporating the temporal characteristics of isovolumic and ejection phases.

Myocardial work (MW) quantification was performed by the software based on the acquired GLS data. This involved computing the segmental shortening rate through strain curve differentiation and multiplying it by the instantaneous left ventricular pressure. The following key indices were automatically derived from this framework:

- GCW (Global Constructive Work): Work from systolic shortening and isovolumic relaxation lengthening.
- GWW (Global Wasted Work): Work from systolic lengthening and isovolumic relaxation shortening.
- GWI (Global Myocardial Work Index): Total work area of the LV PSL (mitral valve closure to opening).
- GWE (Global Myocardial Work Efficiency): Efficiency ratio: GCW/(GCW + GWW).

To mitigate multicollinearity interference, we adopted GWE, identified as the optimal parameter in our previous investigation [17], as the representative parameter of left ventricular myocardial work in the current study.

### 2.6. Variability Analysis

Measurement reliability was assessed in a randomly selected subset (*n* = 15). For intra-observer analysis, a blinded observer repeated measurements at 1-week intervals. Interobserver variability was evaluated by two independent analysts masked to clinical data and each other’s assessments. Reliability was quantified using intraclass correlation coefficients (ICC).

### 2.7. Models

Three distinct predictive models were developed for coronary lesion risk stratification:

(a) clinical parameter-based (Model A);
(b) global myocardial work efficiency-derived (Model B);
(c) a composite clinical–GWE model (Model C).

The models were constructed employing binary logistic regression with backward stepwise selection. Their performance was assessed by sensitivity, specificity, accuracy, Youden’s index, and the area under the receiver operating characteristic curve (AUC), followed by external validation using the validation set. To identify the most clinically beneficial model, decision curve analysis (DCA) was performed. The optimal model determined by DCA was then used to generate the final nomogram.

### 2.8. Statistical Analysis

All statistical analyses were performed on SPSS version 25.0 (Chicago, IL, USA), MedCalc (Version 20.0, Ostend, Belgium), and R 4.4.2 (R Foundation, Vienna, Austria). The normality of continuous variables was verified with the Shapiro–Wilk test. Intergroup comparisons were conducted using the independent Student’s *t*-test for normally distributed data (presented as mean ± SD), the Mann–Whitney U test for non-normally distributed data (presented as median [25th and 75th percentiles]), and the chi-square test for categorical variables (presented as *n* [%]). Univariate and multivariable analyses were used to identify variables that could predict the high-risk group. Variables significantly associated with the high-risk group in univariate analysis (*p* < 0.05) were included in a multivariable logistic regression model. DeLong’s test was used for ROC curve comparisons. A *p*-value < 0.05 (two-tailed) was considered statistically significant.

## 3. Results

### 3.1. Baseline Characteristics

Baseline characteristics of the study population are presented in Table 1. In total, this study included 332 eligible patients (training set: *n* = 181; external validation set: *n* = 151) with a mean age of 56.9 ± 9.5 years. Among them, 158 (47.6%) were male, with 88 males (48.6%) in the training cohort and 70 males (46.4%) in the external validation cohort. The training and external validation cohorts were well-matched overall, with the exception of a significant difference in the incidence of hypertension (*p* = 0.04).

The clinical, laboratory, and echocardiographic parameters of the training set were summarized in Table 2. According to GS tertiles, patients were classified into low-GS, moderate-GS, and high-GS groups, with GSs of ≤20, 20–54, and >54, respectively. The present study included 121 patients (66.9%) with low–moderate GSs and 60 patients (33.1%) with high GSs. Compared to the low-to-moderate GS group, patients in the high-GS group exhibited significantly elevated levels of LP-A, Scr, LP-PLA2, and NT-proBNP, along with a reduced GWE (all *p* < 0.05).

### 3.2. Logistic Regression Analysis

Univariate regression analysis showed that LP-A, Scr, LP-PLA2, NT-proBNP, and GWE were significantly associated with high GS. Subsequent multivariable regression analysis confirmed that Scr, LP-PLA2, NT-proBNP, and GWE remained independent factors for CAD risk stratification (Table 3). These four independent predictors were incorporated into a predictive nomogram.

### 3.3. Predictive Performance of Developed Models

Three distinct predictive models were developed in this study: Model A (clinical biomarkers including Scr, LP-PLA2, and NT-proBNP); Model B (myocardial work parameter)—GWE; Model C (Integrated Model)—Combined Model A and Model B.

**Figure 2:**
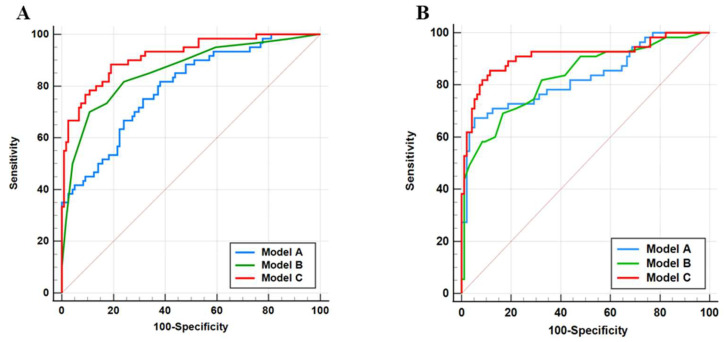
Receiver operating characteristic (ROC) curves in both training (**A**) and validation sets (**B**). The diagonal line indicates the reference line of no discrimination (AUC = 0.5). Method A, Method B, and Method C represent the modeling methods of clinical biomarkers (Scr, LP-PLA2, and NT-proBNP), myocardial work parameter (GWE), and the combined model (Scr + LP-PLA2 + NT-proBNP + GWE).

The discriminative capacity for identifying severe coronary lesions (high-GS) was quantitatively assessed through receiver operating characteristic (ROC) analysis (Figure 2). As presented in Table 4, the AUC values demonstrated progressive improvement across the model hierarchy: Model A achieved AUCs of 0.793 (training set) and 0.827 (validation set), Model B showed AUCs of 0.858 (training set) and 0.831 (validation set), while the integrated Model C yielded the highest discriminative performance with AUCs of 0.916 (training set) and 0.911 (validation set). Pairwise comparisons using DeLong’s test confirmed Model C’s statistical superiority over Model A (training set: *p* < 0.001; validation set: *p* = 0.01) and Model B (training set: *p* = 0.02; validation set: *p* < 0.001).

The integrated Model C exhibited optimal diagnostic performance across both cohorts, demonstrating 88.33% sensitivity and 80.99% specificity (Youden index = 0.693) in the training set, which was further validated with 85.45% sensitivity and 88.54% specificity (Youden index = 0.740) in the external validation set, suggesting its potential applicability for severe coronary artery disease risk stratification.

### 3.4. DCA Curves

**Figure 3:**
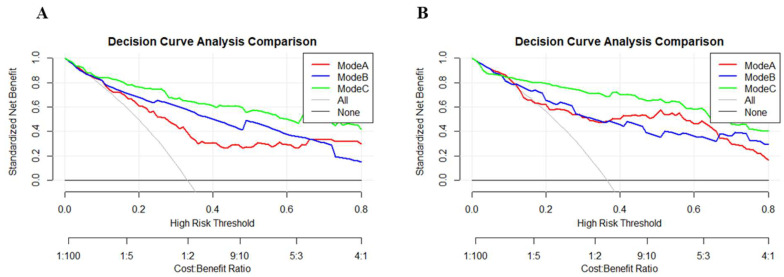
Decision curve analysis (DCA) of the three models in predicting high-GS. (**A**) Training set. (**B**) Validation set.

The clinical utility of the models was assessed using DCA, which demonstrated that the combined model (Model C) offered superior clinical utility (Figure 3). Across the threshold probability range of 0–0.8, Model C demonstrated higher clinical net benefit compared to both Model A and Model B, indicating significant advantages in clinical practice.

### 3.5. Nomogram Construction

**Figure 4:**
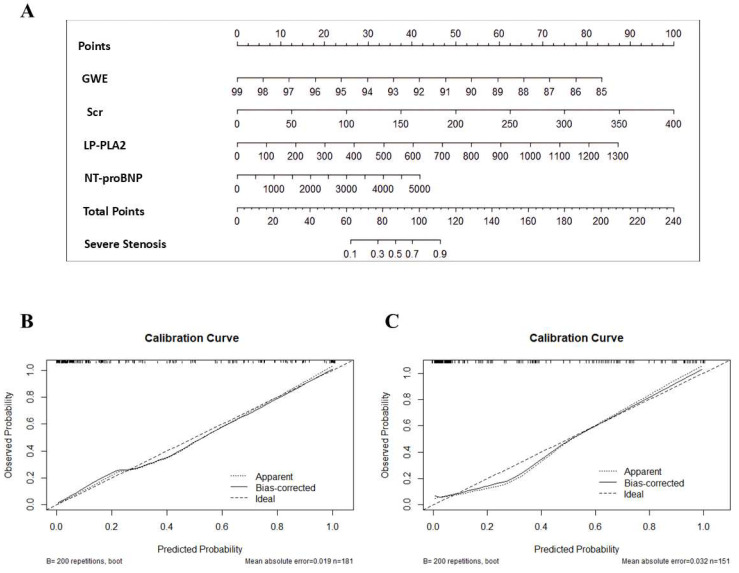
Nomogram combining GWE, Scr, LP-PLA2, and NT-proBNP. (**A**) Nomogram plot. (**B**) Calibration graph for the training set. (**C**) Calibration graph for the validation set. GWE, global work efficiency; Scr, serum creatinine; LP-PLA2, lipoprotein phospholipase A2; NT-proBNP, N-terminal pro brain natriuretic peptide.

An integrative nomogram was developed by incorporating all identified independent risk factors, including Scr, LP-PLA2, NT-proBNP, and GWE for predicting severe coronary artery disease (high-GS) (Figure 4). Each variable in the nomogram was assigned a risk score on the point scale. We were able to calculate the total risk points to estimate the risk of severe coronary artery disease according to the severe stenosis probability scale in the nomogram.

The calibration curves demonstrated good agreement between predicted and observed probabilities in both the training set (mean absolute error, MAE = 1.9%; mean squared error, MSE = 0.00049) and external validation set (MAE = 3.2%; MSE = 0.00157). Notably, 90% of predictions exhibited errors below 3.7% and 6.4% in the training and external validation sets, respectively, indicating reliable performance (Figure 4).

### 3.6. Inter- and Intra-Observer Variability

The inter- and intra-observer variability for GWE is summarized in Supplementary Table S2. Both inter- and intra-observer analyses demonstrated good repeatability, with intraclass correlation coefficient (ICC) values consistently exceeding 0.80.

## 4. Discussion

The present study developed and validated a nomogram integrating GWE with established clinical biomarkers for non-invasive assessment of severe coronary stenosis in stable CAD patients without RWMA. We demonstrate that the combined model (Model C) exhibited better discriminative ability (AUC = 0.916) compared to clinical (Model A, AUC = 0.793) or GWE-only models (Model B, AUC = 0.858), with good calibration (mean absolute error 1.9%). These results suggest that myocardial work analysis, when combined with systemic biomarkers, provides a robust tool for identifying high-risk patients who may benefit from invasive evaluation.

### 4.1. Clinical Implications

Current noninvasive strategies for CAD detection remain limited in patients with preserved LVEF and no RWMA. Our findings demonstrated that GWE, a load-independent measure of myocardial efficiency, significantly enhances risk stratification when integrated with biomarkers. The nomogram’s high sensitivity (88.3%) and specificity (81.0%) at optimal thresholds suggest its potential as a gatekeeper for coronary angiography. Decision curve analysis further supports the clinical utility of Model C, showing consistent net benefit across a wide range of threshold probabilities. This result implies that using the nomogram to guide referral for angiography could reduce unnecessary procedures while appropriately escalating care for high-risk individuals.

Previous studies have developed prediction models based on clinical factors and biomarkers for obstructive CAD, demonstrating acceptable accuracy [21,22,23]. Recently, Liu et al. developed a rest-only single-photon emission computed tomography myocardial perfusion imaging (SPECT MPI)-based nomogram incorporating clinical risk factors to predict obstructive CAD in suspected CAD patients [24]. The model showed competent discrimination (AUC = 0.795) and clinical applicability via DCA. Our integrated model combining established clinical indicators with myocardial work parameter demonstrates good predictive performance (AUC = 0.916), representing a substantial advancement in noninvasive CAD risk stratification.

Recently, computed tomography coronary angiography (CT-CA), particularly when coupled with computed tomography-derived fractional flow reserve (CT-FFR), has established high diagnostic accuracy (AUC > 0.80) for detecting anatomically and functionally significant CAD [25,26,27]. In this study, our echocardiography-based nomogram is not proposed as a replacement for CT-CA in the initial diagnostic evaluation of stable chest pain. Instead, its potential clinical utility may be situated within several specific situations: as an accessible, low-cost, and radiation-free adjunct in patients already undergoing echocardiography for other clinical indications; in settings where CT-CA is contraindicated, unavailable, or yields diagnostically limited images (e.g., due to high coronary artery calcium scores or arrhythmia); or as a complementary tool that provides independent data on myocardial function (via GWE). The application of this nomogram during or immediately following a routine echocardiographic examination could help identify high-risk individuals, thereby prompting further definitive investigation with CT-CA or direct referral to invasive coronary angiography.

### 4.2. Mechanistic Insights

The good predictive performance of GWE fundamentally reflects the pathophysiological cascade of ischemic cardiomyopathy. While RWMA may be absent in early-stage disease, coronary stenosis already compromises myocardial oxygen supply, resulting in mechano-energetic uncoupling during both systolic contraction and diastolic relaxation [16]. This mechanistic framework gains further support from metabolic imaging studies demonstrating a strong correlation (r = 0.82, *p* < 0.001) between myocardial work parameters and regional glucose metabolism quantified by ^18^F-FDG PET [9]. The pressure–strain loop area, as the cornerstone of GWE calculation, inherently encapsulates myocardial oxygen demand and metabolic efficiency, explaining its sensitivity for detecting subclinical ischemia through simultaneous assessment of mechanical dysfunction and metabolic disturbance.

The pathophysiological superiority of GWE is further amplified when integrated with biomarkers that capture distinct facets of the ischemic cascade. This multi-parametric approach mirrors the natural history of CAD progression: from molecular-level plaque activity to end-organ mechanical dysfunction. The integrated model’s robustness stems from its multi-tiered biomarker synergy: (1) LP-PLA2, as a specific marker of plaque inflammation, identifies vulnerable coronary lesions that may precipitate oxygen supply–demand mismatch [28]; (2) NT-proBNP quantifies the resultant ventricular wall stress [29]; (3) Scr reflects renal function and systemic metabolic homeostasis, with elevated levels altering myocardial energy substrate utilization [30]; and (4) GWE directly measures the resultant mechano-metabolic inefficiency.

Additionally, the integration of myocardial work analysis into stress echocardiography presents a compelling avenue for research. During stress, the mismatch between myocardial oxygen supply and demand is amplified. Assessing the dynamic changes in GWE or regional work parameters from rest to peak stress could potentially provide a highly sensitive, load-independent measure of inducible ischemia, possibly overcoming some limitations of visual wall motion analysis. Future studies should investigate whether stress-induced abnormalities in myocardial work parameters can improve the detection of functionally significant stenoses (as defined by FFR) compared to conventional stress echocardiography. The subsequent development of prognostic models based on stress myocardial work could further refine risk stratification.

### 4.3. Limitations

Our study has several limitations that should be acknowledged. First, we acknowledge that our model predicts anatomical severity (Gensini score) rather than functional ischemia or revascularization need. Although high anatomical burden often correlates with ischemic risk, our study did not validate the model against functional tests (e.g., FFR) or clinical outcomes. Thus, the model serves as an anatomical risk-stratification tool to identify patients who may warrant further functional or invasive evaluation. Prospective validation against ischemia and clinical endpoints is needed to define its role in patient management. Second, detailed data on angina severity (e.g., CCS class) and the specific results of non-invasive stress tests (e.g., stress echocardiography) that led to the CAG referral were not systematically collected in our retrospective cohort. This limits our ability to precisely define the nomogram’s position within the existing diagnostic algorithm for stable CAD, and to understand its potential additive value to the current standard-of-care assessment. Third, GWE calculation relies on brachial pressure estimates, which may not fully reflect left ventricular afterload. Fourth, the single-center retrospective design may introduce selection bias, and the exclusion of RWMA patients limits generalizability to the broader CAD population. External validation in multicenter cohorts is required before clinical implementation.

## 5. Conclusions

This study proposes a clinically practical nomogram combining myocardial work efficiency and biomarkers to identify severe coronary stenosis in stable CAD patients without RWMA. With favorable discriminative power and calibration accuracy, this tool may refine preoperative risk stratification and optimize the use of invasive diagnostics.

## Figures and Tables

**Table 1:** Baseline characteristics of the study population.

Variable	All Cohort (*n* = 332)	Training Set (*n* = 181)	Validation Set (*n* = 151)	*p* Value
Age (years)	56.9 ± 9.5	56.0 ± 10.6	57.8 ± 8.0	0.09
Male sex, *n* (%)	158 (47.6%)	88 (48.6%)	70 (46.4%)	0.68
Heart rate (bpm)	77.5 (69.0, 86.8)	79.0 (70.0, 87.5)	76.0 (68.0, 86.0)	0.13
Body mass index (kg/m^2^)	24.7 (23.4, 25.8)	24.8 (23.7, 25.9)	24.5 (23.1, 25.8)	0.15
SBP (mmHg)	138.0 (128.0, 148.0)	139.0 (128.0, 150.0)	136.0 (127.0, 146.0)	0.10
Hypertension, *n* (%)	137 (41.3%)	84 (46.4%)	53 (35.0%)	0.04 *
Hyperlipidemia, *n* (%)	115 (34.6%)	65 (35.9%)	50 (33.1%)	0.56
Diabetes mellitus, *n* (%)	116 (35.8%)	59 (32.6%)	57 (37.7%)	0.33
Current smokers, *n* (%)	126 (38.0%)	72 (39.8%)	54 (35.8%)	0.45
Gensini score	34.5 (14.0, 66.8)	32.0 (14.5, 68.0)	37.0 (13.0, 65.0)	0.75
LVEF (%)	63.0 (59.2, 65.8)	63.0 (59.0, 65.0)	63.0 (60.0, 66.0)	0.13
Medications				
β-blockers	44 (13.3%)	26 (14.4%)	18 (11.9%)	0.51
Statins	93 (28.0%)	53 (29.2%)	40 (26.5%)	0.57
ARBs	41 (12.3%)	25 (13.8%)	16 (10.6%)	0.38
ACE inhibitors	38 (11.4%)	23 (12.7%)	15 (9.9%)	0.43
Calcium channel blockers	98 (29.5%)	55 (30.3%)	43 (28.4%)	0.70
Diuretic	25 (7.5%)	15 (8.3%)	10 (6.6%)	0.57

CAD, coronary artery disease; SBP, systolic blood pressure; DBP, diastolic blood pressure; ARB, angiotensin II receptor blocker; ACE, angiotensin-converting-enzyme. Data are expressed as mean ± SD, median (p25, p75), or as number (percentage). * Significantly different (*p* < 0.05) compared with the validation set.

**Table 2:** Clinical, laboratory, and echocardiographic parameters of the training cohort.

Variable	Training Set (*n* = 181)	Low–Moderate GS (*n* = 121)	High-GS (*n* = 60)
Age (years)	56.0 ± 10.6	55.9 ± 10.9	56.4 ± 10.0
Male sex, *n* (%)	88 (48.6%)	57 (47.1%)	31 (51.7%)
Heart rate (bpm)	79.0 ± 10.7	78.2 ± 10.2	80.4 ± 11.4
Body mass index (kg/m^2^)	24.8 (23.7, 25.9)	24.8 (23.4, 25.9)	24.7 (23.5, 25.9)
SBP (mmHg)	139.1 ± 11.9	138.9 ± 12.5	139.7 ± 10.6
Current smokers, *n* (%)	72(39.8%)	44(36.4%)	28 (46.7%)
TC (mmol/L)	4.3 (3.6, 5.2)	4.2 (3.5, 5.0)	4.7 (3.6, 5.4)
TG (mmol/L)	1.4 (1.0, 1.9)	1.4 (1.0, 1.9)	1.3 (0.9, 1.9)
HDL (mmol/L)	1.2 ± 0.3	1.2 ± 0.3	1.2 ± 0.4
LDL (mmol/L)	2.1 ± 0.7	2.0 ± 0.7	2.1 ± 0.7
LP-A (mg/L)	104.0 (50.0, 256.0)	74.0 (45.0, 166.0)	256 (109.0, 335.2) *
Scr (umol/L)	82.0 (61.5, 107.0)	72.0 (58.0, 96.0)	101 (82.2, 168.5) *
BG (mmol/L)	5.5 (4.9, 6.7)	5.4 (4.7, 6.7)	5.6 (4.9, 6.8)
LP-PLA2 (ng/mL)	154.5 (102.1, 229.8)	144.3 (84.1, 198.1)	205.2 (114.6, 293.5) *
NT-proBNP (pg/mL)	268.4 (166.2, 355.2)	249.5 (156.1, 336.5)	308.8 (208.4, 1097.1) *
APOA1 (g/L)	1.3 (1.1, 1.6)	1.3 (1.1, 1.5)	1.4 (1.1, 1.6)
APOB (g/L)	0.8 (0.7, 1.0)	0.8 (0.7, 1.0)	0.9 (0.7, 1.1)
SV/BSA (mL/m^2^)	39.8 (35.5, 43.5)	38.7 (34.4, 43.5)	40.0 (37.4, 43.5)
LVEF (%)	63.0 (59.0, 65.0)	63.0 (60.0, 65.5)	63.0 (57.3, 65.0)
GWE (%)	93.0 (90.0, 96.0)	95.0 (93.0, 96.5)	89.5 (88.0, 92.0) *

Abbreviations: SBP, systolic blood pressure; TC, total cholesterol; TG, triacylglycerol; HDL, high-density lipoprotein; LDL, low-density lipoprotein; LP-A, lipoprotein A; Scr, serum creatinine; BG, blood glucose; LP-PLA2, lipoprotein phospholipase A2; NT-proBNP, N-terminal pro brain natriuretic peptide; APOA1, apolipoprotein A1; APOB, apolipoprotein B. SV, stroke volume; BSA, body surface area; GWE, global myocardial work efficiency. Data are expressed as mean ± SD, median (25th and 75th percentiles), or as number (percentage). * Significantly different (*p* < 0.05) compared with the low–moderate GS group.

**Table 3:** Univariate and multivariable logistic regression analyses of variables predictive of high GS in non-RWMA patients.

	Univariable Analysis		Multivariable Analysis		
Variable	OR (95% CI)	*p*	OR (95% CI)	Beta (SE)	*p*
Age (years)	1.005 (0.976, 1.035)	0.732			
Sex	1.200 (0.646, 2.230)	0.564			
HR (bpm)	1.020 (0.990, 1.050)	0.196			
BMI (kg/m^2^)	1.165(0.931,1.457)	0.183			
SBP (mmHg)	1.006 (0.980, 1.033)	0.654			
Current smokers	1.531(0.817,2.869)	0.184			
GWE	0.596 (0.513, 0.693)	<0.001	0.612 (0.509, 0.736)	−0.492 (0.094)	<0.001
TC (mmol/L)	1.271 (0.958, 1.687)	0.097			
TG (mmol/L)	0.891 (0.654, 1.215)	0.467			
HDL (mmol/L)	0.893 (0.342, 2.331)	0.818			
LDL (mmol/L)	1.129 (0.709, 1.798)	0.608			
LP-A (mg/L)	1.007 (1.004, 1.009)	<0.001	1.003 (0.999, 1.007)	0.003 (0.002)	0.094
Scr (umol/L)	1.034 (1.020, 1.047)	<0.001	1.021 (1.002, 1.040)	0.020 (0.01)	0.034
BG (mmol/L)	0.984 (0.828, 1.170)	0.859			
LP-PLA2 (ng/mL)	1.007 (1.004, 1.011)	<0.001	1.006 (1.000, 1.012)	0.006 (0.003)	0.043
NT-proBNP (pg/mL)	1.001 (1.000, 1.002)	0.004	1.001 (1.000, 1.001)	0.001 (0.0004)	0.049
APOA1 (g/L)	2.498 (0.734, 8.507)	0.143			
APOB (g/L)	3.425 (0.781, 15.025)	0.103			

HR, heart rate; BMI, body mass index; SBP, systolic blood pressure; GWE, global myocardial work efficiency; TC, total cholesterol; TG, triacylglycerol; HDL, high-density lipoprotein; LDL, low-density lipoprotein; LP-A, lipoprotein A; Scr, serum creatinine; BG, blood glucose; LP-PLA2, lipoprotein phospholipase A2; NT-proBNP, N-terminal pro brain natriuretic peptide; APOA1, apolipoprotein A1; APOB, apolipoprotein B; SE, standard error.

**Table 4:** Receiver operating characteristic curve analysis for the detection of high-GS CAD.

Training Set						Validation Set				
	AUC	AUC 95%CI	SEN (%)	SPE (%)	Y	AUC	AUC 95%CI	SEN (%)	SPE (%)	Y
Model A	0.793	0.727–0.850	81.67	61.98	0.437	0.827	0.758–0.884	67.27	94.79	0.620
Model B	0.858	0.799–0.906	70.0	89.26	0.592	0.831	0.761–0.887	69.09	83.33	0.524
Model C	0.916	0.866–0.952	88.33	80.99	0.693	0.911	0.853–0.951	85.45	88.54	0.740

AUC, area under the curve; SEN, sensitivity; SPE, specificity; Y, Youden index. Model A: clinical indicators (Scr + LP-PLA2 + NT-proBNP); Model B: myocardial work parameter (GWE); Model C: combined model (Model A + Model B).

## Data Availability

The data presented in this study are available on request from the corresponding author due to privacy restrictions.

## Supplementary Materials

The following supporting information can be downloaded at: https://www.mdpi.com/article/10.3390/diagnostics16040570/s1. Table S1: Gensini scoring system; Table S2: ICCs for intra- and interobserver variability for GWE.
