# Supplementary material for: Global Myocardial Work-Derived Nomogram for Coronary Stenosis Assessment in Stable Coronary Artery Disease: Development and External Validation
Source: Diagnostics (Basel). 2026 Feb 13;16(4):570. doi: 10.3390/diagnostics16040570 (PMC12939228; doi:10.3390/diagnostics16040570)
Supplement: Supplementary file 1 [file diagnostics-16-00570-s001.zip › diagnostics-4107282-supplementary.pdf]

## Supplementary Table S1 Gensini scoring system

**STEP 1 Calculation of the severity score for each lesion ≥25% and adjustment for total occlusions or 99% obstructive lesions receiving collaterals**

| Degree of stenosis | Receiving collaterals | Adjustment for collaterals | Severity score |
|---|---|---|---|
| 1%-25% | - | 0 | 1 |
| 26%-50% | - | 0 | 2 |
| 51%-75% | - | 0 | 4 |
| 76%-90% | - | 0 | 8 |
| 91%-99% | no | 0 | 16 |
| 99% | yes | -8 | 8 |
| 100% | no | 0 | 32 |
| 100% | Yes, and normal source vessel | -16 | 32-16=16 |
| 100% | Yes, and 25% stenosis source vessel | -12 | 32-12=20 |
| 100% | Yes, and 50% stenosis source vessel | -8 | 32-8=24 |
| 100% | Yes, and 75% stenosis source vessel | -4 | 32-4=28 |
| 100% | Yes, and 90% stenosis source vessel | -2 | 32-2=30 |
| 100% | Yes, and 99% stenosis source vessel | -1 | 32-1=31 |

**STEP 2 A multiplying factor is applied to each lesion score based upon its location in the coronary tree**

| Segment | Right Dominance | Left Dominance |
|---|---|---|
| RCA proximal | 1 | 1 |
| RCA mid | 1 | 1 |
| RCA distal | 1 | 1 |
| PDA | 1 | 1 |
| PLB | 0.5 | 0.5 |
| LM | 5 | 5 |
| LAD proximal | 2.5 | 2.5 |
| LAD mid | 1.5 | 1.5 |
| LAD apical | 1 | 1 |
| 1st Diagonal | 1 | 1 |
| 2nd Diagonal | 0.5 | 0.5 |
| LCX proximal | 2.5 | 3.5 |
| LCX mid | 1 | 2 |
| LCX distal | 1 | 2 |
| Obtuse Marginal | 1 | 1 |

RCA, right coronary artery; PDA, posterior descending artery; PLB, posterolateral branch; LM, left main coronary artery; LAD, left anterior descending; LCX, left circumflex artery.

The Gensini score (GS) was calculated through a two-step process: (1) assigning standardized severity scores to individual coronary stenoses, with adjusted weighting for total occlusions or 99% obstructive lesions supported by collateral circulation, followed by (2) multiplication by a location-specific coefficient reflecting the hemodynamic significance of each lesion's position within the coronary arterial tree

**Supplementary Table S2 ICCs for intra- and interobserver variability for GWE**

| Variable | Cohort | Inter-observer variability | | Intra-observer variability | |
|---|---|---|---|---|---|
| | | ICC | 95%CI | ICC | 95%CI |
| GWE | Training set | 0.910 | 0.756-0.969 | 0.861 | 0.637-0.951 |
| | Validation set | 0.909 | 0.751-0.968 | 0.841 | 0.592-0.944 |

ICC, intra-class correlation coefficient; CI, confidence interval; GWE, global myocardial work efficiency.
